# Prevalence of tongue lesions in the Indian population

**DOI:** 10.4317/jced.51102

**Published:** 2013-07-01

**Authors:** Santosh Patil, Sumita Kaswan, Farzan Rahman, Bharati Doni

**Affiliations:** 1 Dept of Oral medicine and radiology. Jodhpur Dental College, Jodhpur National University, Jodhpur (Raj). India; 2 Dept of Conservative Dentistry and Endodontics, Jodhpur Dental College, Jodhpur National University, Jodhpur (Raj). India; 3 Dept of Oral Pathology and Microbiology. Jaipur Dental College and Hospital. Jaipur (Raj). India; 4 Dept of Oral medicine and radiology. NIMS. Jaipur (Raj). India

## Abstract

Objective: Tongue lesions are a health concern for the dental practitioners and the patients as they constitute a significant proportion of oral mucosal lesions. The aim of the present study was to determine the prevalence of various tongue lesions in the Indian population. Material and methods: 4926 patients attending the Department of Oral Medicine and Radiology were examined for the presence of various tongue lesions during the period from October, 2010 to September, 2012. The age of the patients ranged from 12-80 years with a mean age of 36.51 years. Results: The prevalence of tongue lesions was 12.07%. The most common lesion diagnosed was coated tongue affecting 28.0% of the subjects, followed by geographic tongue (16.4%), fissured tongue (14.9%) and depapillated tongue (11.5%). Males were more frequently affected than females. The most common systemic condition observed in the patients with tongue lesions was anaemia (189), followed by hypertension (47) and diabetes mellitus (38). Conclusion: The high prevalence necessitates adequate awareness of the various tongue lesions in the general population. The dental clinicians should also be knowledgeable about the etiopathogenesis, clinical presentation, diagnosis, and treatment of these lesions.

** Key words:**Tongue lesions, prevalence, Indian population, coated tongue.

## Introduction

Tongue lesions constitute a considerable proportion of the oral lesions, which are of prime concern when considering oral and general health of an individual (1). It performs various functions such as taste, swallowing, speech, suckling, general sensations and helps in development of the jaw (2), which may be affected by changes in the oral conditions. Various epidemiological studies have been done around the globe reporting the prevalence of tongue lesions in different populations (3-6). Differences have been reported due to variations in the ethinicity, geographical differences, design of the study, diagnostic criteria used for the study, and gender variations in the study samples (7,8). Majority of the lesions are supposed to be developmental anomalies and are rarely discovered by the patient (9). These are usually identified during routine dental checkup. Most of the lesions are due to local etiological factors. Though, they have also been shown to present in association with other pathological conditions and systemic diseases. Hence, the early diagnosis can aid in the identification of these conditions. The lesions can be limited to the tongue or may involve adjacent oral mucosal structures (10).

The studies done till date provide a base-line data for dental practitioners and oral health care workers for the treatment planning and patient education. No such study has been done in the Indian subcontinent. The present study was designed to study the prevalence of the common tongue lesions in the Indian population and the presence of various systemic conditions in association with these lesions.

## Material and Methods

4926 patients attending the Department of Oral Medicine and Radiology, Jodhpur Dental College General Hospital, were examined for the presence of various tongue lesions in the period from October, 2010 to September, 2012. All the patients of the age range from 12-80 years attending for routine dental checkup were examined for various tongue lesions. Ethical clearance from the Institutional Ethical Committee was obtained. A written informed consent was obtained from the patient. The clinical examination of the oral cavity and tongue was done following the WHO guidelines (11), under artificial illumination on a dental chair, using a mouth mirror. The tongue was examined for any surface changes, specific lesions, size and movements. Lymph nodes were also examined. Very few of the patients were aware of the lesions present. Most of the patients were asymptomatic. Those with symptoms had complaints such as burning sensation of the tongue, painful ulcerations, difficulty in speech, altered taste sensations, appearance of the tongue and difficulty in movements, in cases with large lesions. None of the patients were under medication for any of the lesions examined. A detailed family and medical history and history in relation with any habits of tobacco/smoking/alcohol was recorded. Histopathological confirmation was required in a few cases to confirm the clinical diagnosis.

## Results

The study comprised of 4926 patients, of which 2597 were males and 2329 were females. The age of the patient ranged from 12-80 years with a mean age of 36.51 years with a standard deviation of 2.69 years ( Table 1). Of the total patients examined, 595 patients were diagnosed with various tongue lesions. The distribution of various lesions is presented in  Table 2. The most common lesion diagnosed in the study sample was coated tongue affecting 28.0% of the subjects (167 cases). Geographic tongue was seen in 98 patients (16.4%), fissured tongue in 89 patients (14.9%), depapillated tongue in 69 patients (11.5%), hairy tongue was seen in 32 patients (5.3%) and traumatic ulcerations was seen in 29 patients (4.8%). Ankyloglossia was seen in 21 patients (3.5%) and macroglossia was seen in 9 patients (1.5%). Leukoplakia was seen in 3.0% of the patients and 3.7% patients were diagnosed with median rhomboid glossitis. Aphthous ulcers were seen in 11 patients. Malignant and benign tumors such as squamous cell carcinoma, fibroma, papilloma and haemangioma were seen in only 10 patients. The prevalence of tongue lesions was 12.07%. Most of the patients were asymptomatic and reported to the out patient department with other dental problems. Very few patients were aware of the lesions. Males were more frequently affected than females ( Table 3). Various systemic conditions were seen in the patients with tongue lesions ( Table 4). The most common systemic condition observed in the patients with tongue lesions was anaemia (189 patients), followed by hypertension (47 patients) and diabetes mellitus (38 patients).

**Table 1:**
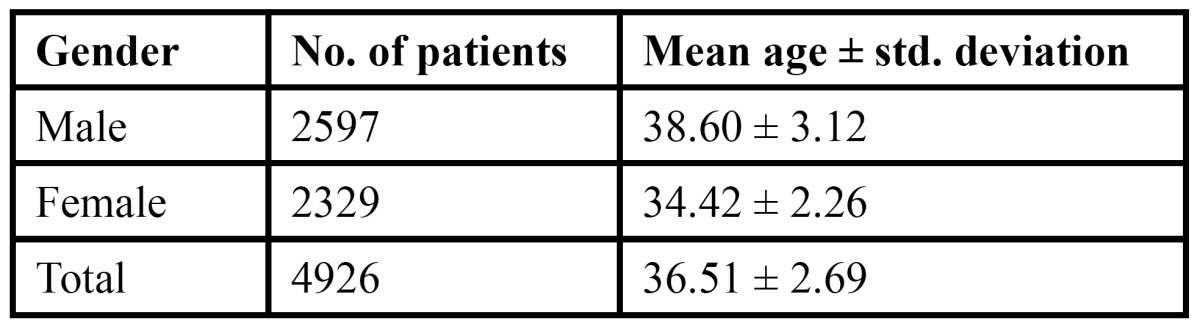
Distribution of patients according to gender with mean age±standard deviation.

**Table 2:**
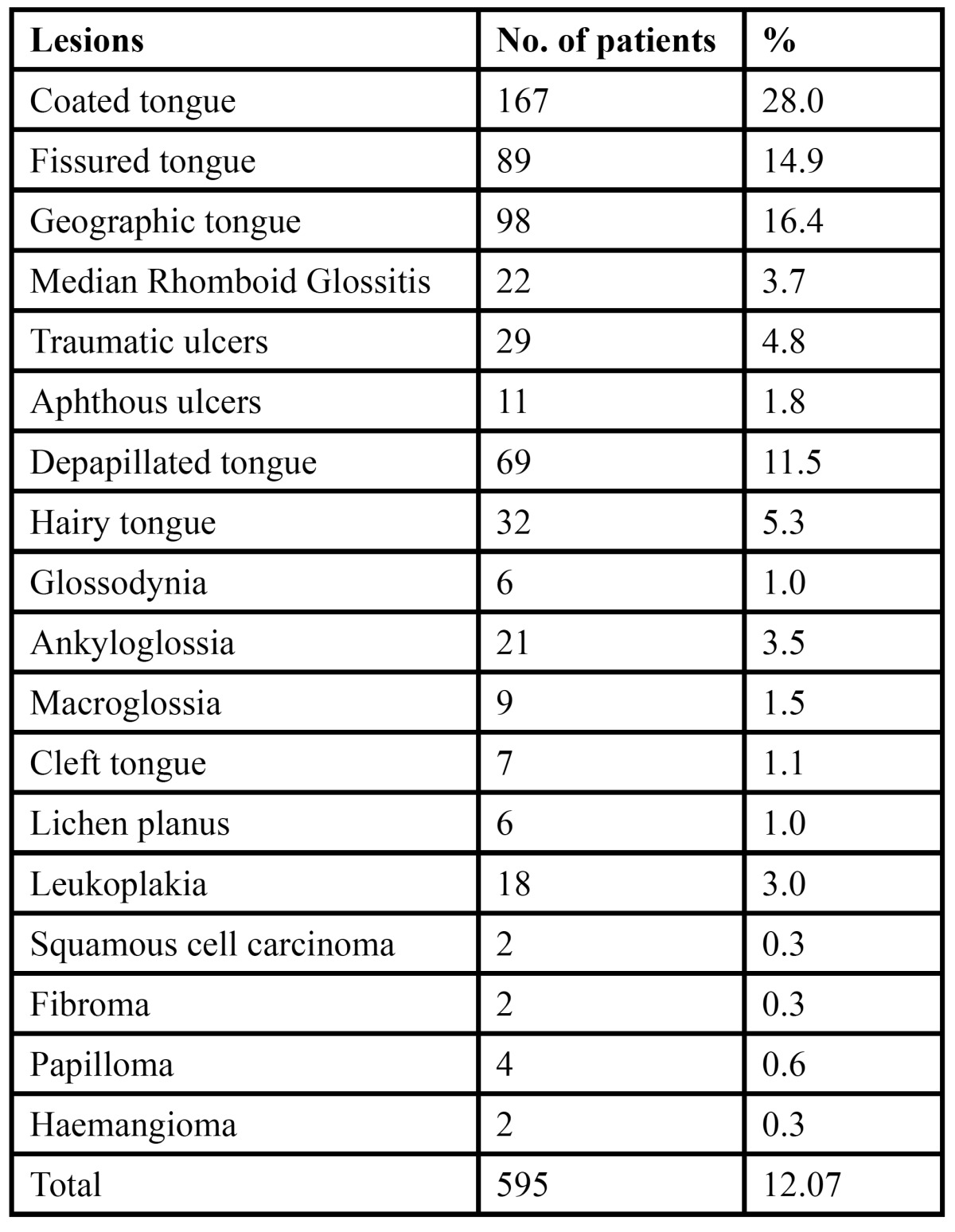
Distribution and prevalence of various tongue lesions.

**Table 3:**
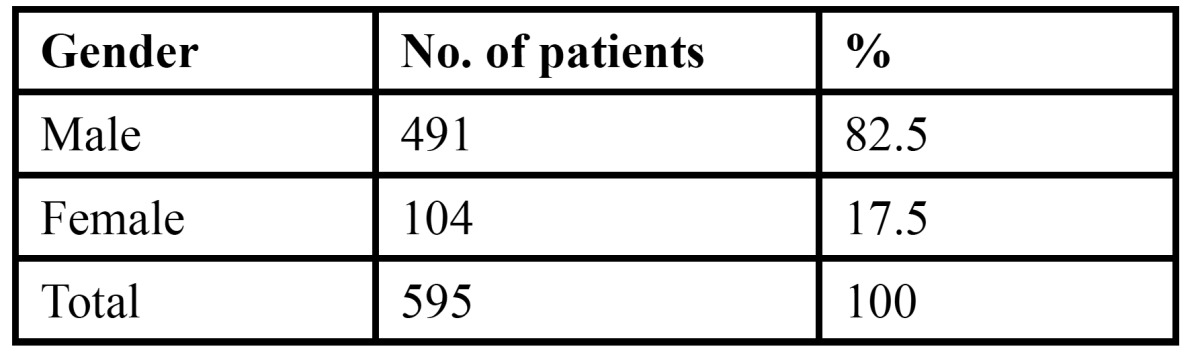
Distribution of tongue lesions according to gender.

**Table 4:**
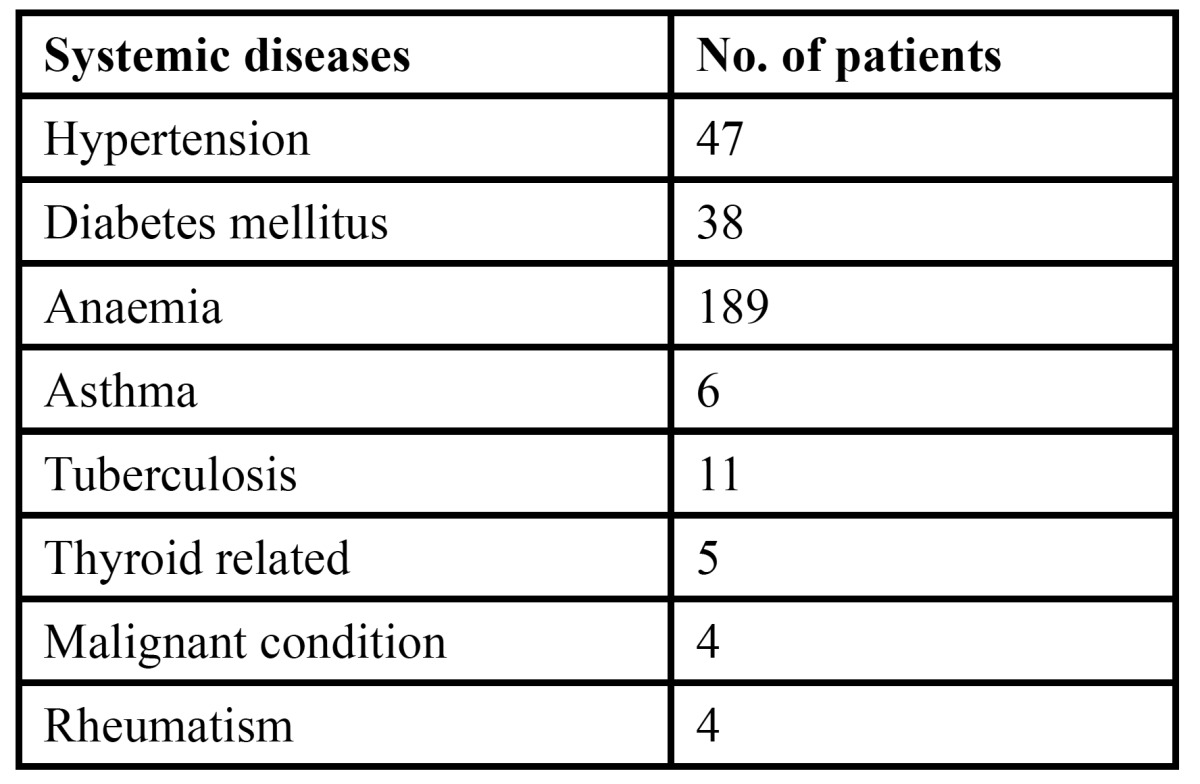
Distribution of systemic diseases in patients with tongue lesions.

## Discussion

Although easily examined, most of tongue lesions can present a diagnostic and therapeutic dilemma for the dental practitioners. Early identification and diagnosis can be done by a thorough history of the lesion, preceding symptoms, and related habits of tobacco/smoking and alcohol. Various epidemiological studies have shown the prevalence of tongue lesions in different parts of the world to be approximately upto 18.5% (5,12).

The prevalence of coated tongue in the present study was the highest (28.0%). This is not in accordance with the previous studies, which showed that fissured tongue was the most common lesion in various populations (1,5,8,10). The prevalence of coated tongue was reported to be 9.2% (5) and 11.0% (8) in studies done by Dar-wazeh et al. in the Jordanian population. Coated tongue was significantly related to smoking (8,13) and hairy tongue (5,14). The prevalence of coated tongue in the present study was much higher than the previous studies. In a similar study in the Turkish population (3) also coated tongue was the most prevalent lesion but the preva-lence was very low (2.1%) when compared to the present study.

The prevalence of geographic tongue in the present study was 16.4%. This was in line with the findings in the Brazilian population and Libyan population which showed a prevalence of 21% and 17.4% respectively (10,15). The prevalence in different populations shows a wide variation. The results of the present study was much higher than 6.8% and 4.8% previously reported in the Jordanian population (5,8), 0.6% in the American population (16) and 1.6% in the South African population (17). The geographic tongue is more common during childhood, though it has been reported in subjects >40 years of age and is seen predominantly in females (7,12). In the present study also females were more commonly affected. Male predominance has been reported by Vörös-Balog et al. (18). Thus, the association of geographic tongue to gender is not consistent. But its association with fissured tongue has been well reported in the literature (18). The wide discrepancy in the results can be due to the transient nature of geographic tongue, differences in the ethnicity and the different clinical criteria used in the study.

Fissured tongue was reported in 89 patients with a prevalence of 14.9%. The prevalence was consistent with previous studies as reported in the literature, but it was not the most frequent lesion as mentioned in these studies (5,8). The Libyan population had an even higher prevalence of 48.4% (10). The Brazilian population also reported the prevalence to be 27.3%, which was higher than the results of the present study (19). The prevalence was quite low among the Saudi population (1.4%) (20) and the Turkish population (21). This lesion has been suggested to be genetically determined. Various contributory factors to the development of fissured tongue include hyposalivation, diabetes mellitus, candidiasis, vitamin B deficiency and lichenoid reactions (10). With advancing age the prevalence of fissured tongue increases. This can be explained by the fact that increasing age is associated with hyposalivation, which is one of the prime contributing factors (8). Fissured tongue has been seen in patients with Down’s syndrome, acromegaly, psoriasis, and Sjögren syndrome. Melkersson-Rosenthal syndrome is characterized by a triad of severe fissuring, relapsing orofacial edema, and facial nerve palsy (9). Most of the patients with fissuring of the tongue present with no symptoms; however symptoms such as soreness with acidic food and beverages may be seen if the fissures are deep. The deep fissures act as reservoir for food particles and accumulate bacteria leading to the inflammation of the tongue.

Tongue depapillation was reported in 69 patients (11.5%). The prevalence in the Libyan population was reported to be 25.6%, which is higher than the results of the present study (10). It is characterized by localized or extensive loss of papillae. It may be associated with burning sensation in some patients. The depapillated areas are patchy. It is commonly seen in patients with nutritional deficiencies, xerostomia, lichenoid reactions, local trauma and candidiasis (1).

Median rhomboid glossitis has been suggested to be either a congenital anomaly or a form of candida infection (9). The prevalence of median rhomboid glossitis in the present study was reported to be 3.7%. A prevalence of 0.6% has been reported in Jordanian and Libyan population (8,10). The present study however, reported a higher prevalence. This lesion improves with the administration of anti-fungal drugs. Some patients may not show any improvement with anti-fungal agents. In such cases the dentist should be able to diagnose the condition and recognize the etiology, so that appropriate treatment can be given to the patient. It is more prevalent in males and in most cases is asymptomatic. Patients may sometimes present with burning sensation and itching (9).

The prevalence of hairy tongue in the present study was reported to be 5.3%. the prevalence varies from 0%-11.3% (3,18). The findings of the present study are in line with those reported in the Jordanian population (5.8%) (8). However, it is quite high when compared to the Libyan population, which reported a prevalence of 4.4% (10). It is manifested as the elongation of the filliform papillae due to accumulation of excess keratin, in response to infections, fever, antibiotics, tobacco and xerostomia. The tongue may appear of different colours. Most patients are asymptomatic but few may complain of oral malador or altered taste perception (9).

Ankyloglossia or tongue-tie is a congenital developmental anomaly that limits tongue protrusion due to an abnormally short lingual frenulum (9). The prevalence of ankyloglossia in various studies has been estimated to be 0.1%-3.7% (22). The present study also reported a prevalence of 3.5%, which is similar to the previous studies. Macroglossia is the abnormal enlargement of the tongue in relation to the jaws and the oral cavity. It is associated with Down’s syndrome, tuberculosis, sarcoidosis, hypothyroiddism, amyloidosis, multiple myeloma, neurofibromatosis, infection and allergic reaction. The prevalence of macroglossia in the present study was 1.5%. It is much higher than that reported in the Turkish population (21).

The prevalence of leukoplakia in the present study was 3.0%, which is much higher than the Libyan population with a prevalence of 0.3% (10). It is most commonly associated with tobacco use and the condition resolves spontaneously with the cessation of the habit. It is a premalignant condition and thus, microscopic analysis and biopsy is recommended (9). Lichen planus was seen in 6 patients with a prevalence of 1.0%. The prevalence in the Libyan population was reported to be 2.2%, which was higher than the present study (10). It is seen com-monly involving the lateral borders of the dorsum of the tongue.

Traumatic ulcerations were seen in 4.8% patients. The prevalence in the Libyan population was reported to be 1.6% (10). They are caused mostly by sudden biting on the tongue. Aphthous ulcers were seen in 1.8% patients, similar to the results of the Libyan population (1.6%) (10). Benign tumors such as fibroma, haemangioma and papilloma were seen in a total of 8 patients. Fibroma is caused due to chronic irritation and can be differentiated by excisional biopsy. Papilloma is a common lesion seen in 1% individuals and is associated with human papilloma virus infection (9). Squamous cell carcinoma was seen in 2 patients with a prevalence of 0.3%. Ulcers of the tongue should be examined carefully to rule out any malignancy.

The significance of the various tongue lesions with systemic conditions should not be overlooked. It may be over emphasized by some due to the non specificity of these lesions associated with these diseases. Most of the patients were asymptomatic and only a few patients reported with associated pain, burning sensations, intolerance to spicy foods, difficulty while eating and speech. Also, only few patients were aware of the lesion and wanted to seek treatment for the same. Due to the high prevalence of these lesions in the general population, dental clinicians should be aware of the clinical appearance, etiology, diagnosis and the required treatment for the lesion.

## Conclusion

The results of the present study are similar to the previous reported studies, but due to the lack of similar studies in the Indian subcontinent, no conclusion can be drawn regarding the exact prevalence in this region. The present study is the first study to report the prevalence of various tongue lesions in India. The present data will provide more information regarding the tongue lesions and may alert the dental clinician regarding any associated underlying systemic conditions. Patients with lesions of unclear etiology can be referred to a specialist and thorough knowledge of the clinical features can be lifesaving in some subjects by early diagnosis and referral.
